# Revisiting Skeletal Muscle Dysfunction and Exercise in Chronic Obstructive Pulmonary Disease: Emerging Significance of Myokines

**DOI:** 10.14336/AD.2023.1125

**Published:** 2023-12-07

**Authors:** Lihua Han, Peijun Li, Qinglan He, Chen Yang, Meiling Jiang, Yingqi Wang, Yuanyuan Cao, Xiaoyu Han, Xiaodan Liu, Weibing Wu

**Affiliations:** ^1^School of Exercise and Health, Shanghai University of Sport, Shanghai, China.; ^2^School of Rehabilitation Science, Shanghai University of Traditional Chinese Medicine, Shanghai, China.

**Keywords:** chronic obstructive pulmonary disease, skeletal muscle dysfunction, myokines, exercise

## Abstract

Skeletal muscle dysfunction (SMD) is the most significant extrapulmonary complication and an independent prognostic indicator in patients with chronic obstructive pulmonary disease (COPD). Myokines, such as interleukin (IL)-6, IL-15, myostatin, irisin, and insulin-like growth factor (IGF)-1, play important roles in skeletal muscle mitochondrial function, protein synthesis and breakdown balance, and regeneration of skeletal muscles in COPD. As the main component of pulmonary rehabilitation, exercise can improve muscle strength, muscle endurance, and exercise capacity in patients with COPD, as well as improve the prognosis of SMD and COPD by regulating the expression levels of myokines. The mechanisms by which exercise regulates myokine levels are related to microRNAs. IGF-1 expression is upregulated by decreasing the expression of miR-1 or miR-29b. Myostatin downregulation and irisin upregulation are associated with increased miR-27a expression and decreased miR-696 expression, respectively. These findings suggest that myokines are potential targets for the prevention and treatment of SMD in COPD. A comprehensive analysis of the role and regulatory mechanisms of myokines can facilitate the development of new exercise-based therapeutic approaches for patients with COPD.

## INTRODUCTION

Chronic obstructive pulmonary disease (COPD) is a heterogeneous lung condition characterized by chronic respiratory symptoms due to airway and/or alveolar (emphysema) abnormalities, which cause persistent, often progressive, airflow obstruction [1]. It has emerged as a growing problem in the 21st century and is one of the top three causes of death worldwide [2]. Skeletal muscle dysfunction (SMD) is the most severe extrapulmonary complication in patients with COPD and has an incidence rate of 15%-55% [3,4]. SMD can occur in the early stages of pulmonary function decline and is manifested by reduced skeletal muscle strength, endurance, and fatigue; it significantly compromises the quality of life of the patients, increases the healthcare burden, and elevates mortality risk. As a result, it is an independent predictor of death [5,6]. In this regard, managing SMD is crucial for enhancing the quality of life and prognosis of patients with COPD. Despite the significance of SMD in COPD, no specific drugs or targeted or personalized treatment strategies are available to improve this condition. The mechanisms underlying SMD have become a major topic of interest in COPD research.

Myokines are proteins secreted by skeletal muscle cells into the circulation where they exert local effects through autocrine, paracrine, or endocrine pathways on surrounding tissues, such as muscle cells and blood vessels within muscles, or systemic effects when released into the bloodstream [7,8]. Myokines, such as interleukin (IL)-6, IL-15, irisin, and myostatin, modulate muscle strength and exercise capacity in patients with COPD [9-12]. Furthermore, irisin is a novel biomarker used to assess physical activity in patients with COPD [13]. A Mendelian randomization analysis revealed that high levels of circulating insulin-like growth factor 1 (IGF-1) are associated with reduced risks of COPD and asthma [14]. Hence, myokines secreted by skeletal muscles play crucial roles in the occurrence and development of COPD [15].

Exercise has emerged as an important non-pharmacological approach to effectively improve the structure and function of skeletal muscles in COPD [16]. It also regulates the gene expression of myokines in skeletal muscles in COPD [17-19]. However, the precise mechanisms of action of myokines underlying the exercise-induced improvement of SMD in COPD remain unclear. Therefore, this study aims to analyze the mechanisms through which exercise affects myokines by exploring their functions in SMD in COPD, as well as to provide new insights into the pathogenesis and treatment of the disease. Therefore, based on the mechanism underlying the effect of myokines on SMD in COPD, this study analyzed the influence of exercise on myokines to provide new insights into the pathogenesis and treatment of the disease.

## SMD AND MYOKINES IN COPD

### SMD in COPD

In COPD, SMD involves alterations in the structure and function of skeletal muscles. The structural changes in skeletal muscles manifest as muscle atrophy, shifts in myofiber composition, mitochondrial damage, and satellite cell (SC) impairment [5,20]. Changes in myofiber composition involve a transition from type I fibers to type II fibers and a decrease in cross-sectional areas (CSAs) of both fiber types, leading to muscle atrophy and reduced muscle strength [21,22]. Mitochondrial damage primarily entails structural and respiratory functional impairments, increased production of mitochondrial reactive oxygen species, decreased adenosine triphosphate (ATP) production, and reduced muscle fatigue resistance [23]. The impairment of SC is mainly characterized by a decreased number of cell nuclei clustered by transcription factor paired box 7 (Pax-7) and compromised SC activation, leading to inhibition of muscle regeneration and muscle contractile function [24]. In summary, structural changes in skeletal muscles contribute to decreased muscle function, thereby exacerbating the exercise capacity and functional abilities of patients [25].

### Myokines in SMD

Myokines are cytokines secreted by skeletal muscle cells that act on various organs, such as bones, brain, and liver, through autocrine, endocrine, or paracrine mechanisms. They can also act locally on muscle tissues to affect muscle structure and function, leading to SMD [26]. Interestingly, the level of myokine secretion depends on the individual’s health and may therefore be more deleterious in patients with various diseases. This suggests that the balance of beneficial and harmful myokines is related to the disease state and health status of patients. Among the first reported myokines, IL-6 is distinct because it is secreted by skeletal muscles during exercise and acts as an energy distribution factor. It downregulates inflammation and promotes muscle energy uptake and contraction [27]. It is a pleiotropic factor, and mounting evidence supports the potential role of IL-6 secreted by skeletal muscles in muscle di-fferentiation and regeneration. Serrano et al. [28] demonstrated that muscle gain in IL-6-knockout mice was blunted in a compensatory hypertrophy model, while exogenous administration of IL-6 rescued these defects. Importantly, IL-6 secreted by myotubes can also promote cell proliferation and differentiation, indicating both autocrine and paracrine roles of IL-6 in muscle growth. Elevated IL-6 levels in the circulation and muscles are associated with severe muscle atrophy under various pathological conditions and result in muscle atrophy [29, 30]. This is because IL-6 can activate a variety of signaling molecules in pathological environments, thereby inducing diseases [31]. Recent experimental and clinical studies have revealed impaired expression of various myokines, including myo-statin, irisin, IGF-1, and IL-15, in individuals with certain diseases. Myostatin, a member of the transforming growth factor-β superfamily, is a known inhibitor of limb muscle growth [32]. *The Lancet* reported that myostatin inhibition led to increased lean mass and potentially improved muscle power in older individuals [33]. A phase II clinical trial demonstrated that the inhibition of myostatin activity improved muscle function in patients with Duchenne muscular dystrophy [34]. Irisin, produced by the fibronectin type III domain-containing protein 5 (Fndc5), is a transmembrane protein [35], which exerts direct effects on mitochondria, protecting mitochondrial function. Guo et al. [9] found that Fndc5/irisin deficiency in aging mice aggravated muscle atrophy, as well as decreased smaller muscle grip strength, muscle weights, and CSA, compared with wild-type mice. Treatment with recombinant irisin protein alleviated sarcopenia and metabolic disorders in aging mice. IGF-1, a well-known growth factor that enhances myofiber regeneration, significantly increased muscle size and absolute force production in the critically ischemic limb [36]. Acting independently or in synergy with IGF-1, IL-15 induces myosin heavy chain synthesis, thereby promoting muscle regeneration [37]. Recent studies have also revealed that IL-15 can stimulate the proliferation of fibro/adipogenic progenitors and inhibit adipogenesis, ultimately promoting fibrosis after muscle damage and facilitating myofiber regeneration [38]. These findings provide a novel perspective on the intricate interplay between myokines and skeletal muscles, presenting a promising therapeutic target for improving SMD in clinical practice. Therefore, myokines deserve careful investigation as preclinical candidate molecules for the treatment of SMD in COPD.

**Table 1:** Differential myokines expression in COPD compared to healthy controls.

Study	Group	Sample size	Degree	Myokines	Tissue	Skeletal muscle structure and function
**Zhang et al., 2022** [15]	CSG	12	/	Myostatin ↑, Fndc5/irisin↓	Quadriceps femoris and gastrocnemius muscle	MyHC of the slow-twitch fibers ↓, MyHC of the fast-twitch fibers↑; grip force ↓
**Silva Lage et al., 2022** [51]	CG HG	43 43	Mild	Irisin↓	Plasma/serum	Irisin was positively correlated with respiratory muscles strength
**Naoki Ijiri Et al., 2015** [13]	CG HG	72 27	Moderate	Irisin↓	Plasma	/
**Zhang et al., 2022** [73]	CG HG	/	Myotubes model	Myostatin↑	Myotubes	Ferroptosis in C2C12 myotubes
**Zhou et al., 2018** [49]	CSG HG	8 8	/	Myostatin↑	Diaphragm	Muscle fiber atrophy, myofilament breakage, and dissolution, mitochondrial swelling, and vacuolization; Myostatin was positively correlated with the diaphragmatic apoptotic and was negatively correlated with the diaphragm mass
**Testelmans et al., 2010** [46]	CG HG	19 13	Severe	Myostatin↑	Diaphragm	Type I% fibers↑, CSA of both type I and type II fibers↓; Diaphragm strength↓
**Man et al., 2010** [48]	CG HG	18 16	Severe	Myostatin↑	Quadriceps	Myostatin was negatively correlated with quadriceps strength, quadriceps endurance, and 6MWD
**Casadevall et al., 2007** [41]	CG HG	25 8	Stable	IL-6↑	External intercostal muscles	IL-6 was positively correlated with the degree of airway obstruction
**Yende et al., 2006** [40]	CG HG	2005 208	Mild Moderate, Severe	IL-6↑	Serum	IL-6 was negatively correlated with quadriceps strength and FEV1% predicted
**Liu et al., 2015** [42]	CSG HG	30 15	/	IL-15↑	Serum, diaphragm, gastrocnemius, and intercostal muscles	The levels of IL-15 in the serum, diaphragm, gastrocnemius, and intercostal muscles were negatively correlated with the weight changes of COPD rats
**Hansen et al., 2013** [52]	CSG	8	/	IGF-1↓ IL-6↑	Gastrocnemius and tibialis anterior	Tibialis anterior and gastrocnemius skeletal muscles atrophy

COPD: chronic obstructive pulmonary disease; CSG: cigarette smoke group; CG: COPD group; CS: cigarette smoke; CSA: cross-sectional area; Fndc5: fibronectin type III domain-containing protein 5; FEV1%: the percentage of forced expiratory volume in one second shared by forced vital capacity; HG: healthy group; IL-6: interleukin-6; IL-15: interleukin-15; IGF-1: insulin-like growth factor-1; MyHC: myosin heavy chain; 6MWD:6-min walk distance; SMM: skeletal muscle mass; ↓: down; ↑: up.

## EMERGING ROLE OF MYOKINES IN COPD

### Myokine dysregulation in COPD

Myokine profiintercostal muscles of patients with COPDles are associated with SMD in COPD. A previous study found that the IL-6 levels are elevated in the skeletal muscles and circulation of patients with COPD; these levels varied with disease severity and are inversely correlated with muscle strength and exercise capacity [39,40]. Casadevall et al. [41] reported increased IL-6 mRNA and protein expression levels in the intercostal muscles of patients with COPD and a positive correlation between IL-6 levels and respiratory muscle dysfunction. In addition, several basic and clinical studies have consistently demonstrated elevated levels of IL-15 and myostatin, and reduced levels of irisin and IGF-1, in the skeletal muscles of patients with COPD [15,41-43]. Liu et al. reported significant increases in IL-15 and myostatin levels in the peripheral and respiratory muscles of COPD rats; these elevated levels had a negative correlation with body weight and muscle function [42,44-46]. In patients with COPD and significant quadriceps weakness, myostatin mRNA transcript levels were three times higher than in the healthy group, showing a negative correlation with quadriceps femoris muscle strength, endurance, and 6-minute walk distance (6MWD) [47,48]. Zhou et al. demonstrated that myostatin overexpression in the diaphragm of COPD rats led to diaphragm apoptosis and atrophy, ultimately resulting in diaphragm weakness and respiratory muscle dysfunction [49]. By contrast, the levels of irisin and IGF-1 were decreased in the serum and skeletal muscles of patients with COPD [50] and had a negative correlation with maximum inspiratory pressure, muscle strength, and 6MWD [51,52]. Furthermore, a retrospective study of 61 patients with COPD demonstrated that serum IGF-1 levels were significantly lower in patients with COPD than in the control group and that the circulating IGF-1 levels were lower in patients with acute exacerbation of COPD than in those with clinically stable COPD [53]. Importantly, irisin not only serves as a novel biomarker associated with physical activity in COPD but also plays a crucial role in regulating the aging process in obesity and diabetes. It exerts positive effects, such as enhancing fatty acid oxidation and heat production, promoting mitochondrial biogenesis, and preventing muscle atrophy in aging and metabolic disease [54]. In summary, dysregulation of myokine expression in the skeletal muscles of patients with COPD is closely associated with SMD in COPD. A comprehensive summary of previous studies is presented in Table 1.

### Potential mechanisms underlying myokine dysregulation in COPD with SMD

### Myokines affect mitochondrial function

Myokines negatively affect mitochondrial function in COPD by decreasing mitochondrial density and respiratory capacity and increasing mitochondrial fission. In particular, IL-6 has been implicated in disrupting mitochondrial dynamics. Fix et al. found that IL-6 increased the expression of mitochondrial fission proteins (Drp-6 and FIS-1) and reduced mitochondrial quality in C2C12 myotubes through the glycoprotein 130 (gp130) transmembrane protein, which is widely expressed in skeletal muscles [55]. Chronic exposure to elevated IL-6 levels can downregulate the expression of mitochondrial complex I/II/IV and cytochrome C oxidase activity in muscle cells through muscle gp130 signaling [56]. Additionally, IL-6 can increase mitochondrial reactive oxygen species production and oxygen consumption in muscle cells by activating the Janus kinase (JAK)/signal transducer and activator of the transcription (STAT) pathway [57]. Recent studies have found that elevated myostatin levels in COPD upregulate the expression of dynamin-related protein 1 (Drp-1), a key protein involved in initiating and promoting mitochondrial division. Tan et al. conducted experiments in C2C12 cells exposed to cigarette smoke (CS) extracts; they reported increased expression of Drp-1 and a significant correlation between myostatin and Drp-1. The Drp-1 expression exhibited a consistent trend with myostatin expression and resulted in reduced mitochondrial damage, ROS production, and cell apoptosis in myostatin siRNA-transfected C2C12 cells [58]. Furthermore, Drysch et al. demonstrated that myostatin knockout inhibited mitochondria-induced cell apoptosis in C2C12 cells [59].

Additionally, the ability of irisin to protect mitochondrial function and ATP synthesis is com-promised by insufficient irisin secretion in COPD. Xin et al. reported that exogenous irisin supplementation increased myocardial ATP production and enhanced the quantity of mitochondrial respiratory complexes I/III/V while simultaneously reducing mitochondrial apoptosis. Furthermore, irisin treatment increased the phos-phorylation level of AMP-dependent protein kinase (AMPK). The protective effects of irisin on cardiac and mitochondrial function in diabetic mice were blocked upon knocking out the AMPK gene, resulting in a significant decrease in ATP production and expression of mitochondrial respiratory complexes I/III/V [60]. Hence, irisin enhances mitochondrial respiratory function through the AMPK pathway. Ye et al. reported that irisin increased mitochondrial autophagy and respiratory capacity in C2C12 skeletal muscle cells through the p38 mitogen-activated protein kinase pathway [61]. Mandal et al. found that serum irisin levels were increased in patients with COPD after treatment with long-acting muscarinic antagonists and that the increased levels were positively correlated with the 6MWD [62]. These findings suggest that insufficient irisin secretion in the skeletal muscles of patients with COPD may hinder its protective effect on mitochondrial function by reducing P-MAPK levels, leading to decreased skeletal muscle exercise capacity and 6MWD. In conclusion, these studies have provided valuable insights that link myokines to mitochondria and enhanced our understanding of the mechanisms underlying mitochondrial dysfunction in skeletal muscles in COPD. The potential therapeutic roles of irisin treatment for improving mitochondrial function warrant further investigation and hold promise for improving SMD in patients with COPD.

### Myokines regulate the balance between skeletal muscle protein synthesis and breakdown

Dysregulation of myokines in skeletal muscles in COPD can lead to an imbalance in muscle protein synthesis and breakdown, thereby exacerbating muscle atrophy. Langen et al. [63] found that abnormally elevated IL-6 levels in the skeletal muscle and circulatory system of patients with COPD interact with pro-inflammatory cytokines, such as tumor necrosis factor α (TNF-α) and IL-1β. Furthermore, they reported that such interaction can led to the activation of the nuclear factor kappa-B signaling pathway and subsequent induction of muscle ring finger-1 (MuRF-1) and muscle atrophy gene-1 (atrogin-1) expression, thereby promoting protein breakdown. IL-6 can promote the release of inflammatory cytokines TNF-α and IL-1β by activating the JAK/STAT3 pathway; moreover, activating the nuclear factor kappa-B pathway can induce skeletal muscle atrophy [64,65]. Elevated myostatin levels can upregulate the expression of MuRF1 and atrogin-1 through the Smad2/3 signaling pathway, further contributing to muscle protein breakdown [66]. Additionally, myostatin negatively regulated irisin expression and secretion by inhibiting the expression of Fndc5 and its upstream peroxisome proliferator-activated receptor γ coactivator-1a (PGC-1α) in skeletal muscles through the Smad signaling pathway [67]. Irisin is a positive regulator of muscle protein synthesis [18], and its reduced level due to the inhibitory effect of myostatin may contribute to muscle atrophy. To explore these mechanisms, Zhang et al. [15] conducted experiments in COPD mice using myostatin inhibitors TEW-7197 (a selective transforming growth factor-β receptor LK4/ALK5 inhibitor) and SIS3 (which inhibits Smad3 phosphorylation) to restore PGC-1α and Fndc5 levels in skeletal muscles. *In vitro* experiments confirmed that irisin mitigated the increased levels of MuRF1 and atrogin1 induced by myostatin and alleviated C2C12 myotube atrophy. In conclusion, dysregulation of myokines, particularly IL-6 and myostatin, plays a significant role in promoting muscle protein breakdown, which leads to skeletal muscle atrophy in COPD.

Downregulation of IGF-1 and IL-15, which are crucial myokines that regulate muscle protein synthesis, significantly impaired normal muscle growth and development, thereby exacerbating skeletal muscle atrophy in COPD [12,68]. In patients with COPD, reduced IGF-1 levels in skeletal muscles led to decreased muscle protein synthesis by inhibiting the muscle hypertrophy pathway IGF-1-phosphoinositide 3-kinase-protein kinase B (Akt) [15,69]. Hansen et al. [52] found that IGF-1 mRNA and protein levels were significantly reduced in the gastrocnemius and tibialis anterior muscles of CS-exposed mice. Furthermore, they reported that the weights of the tibialis anterior and gastrocnemius muscles were significantly reduced by 10.3% and 9.9%, respectively, but the levels of systemic plasma markers of inflammation (C-reactive protein, IL-6, and TNF-α) were not altered. Hence, IGF-1 leads to muscle atrophy by downregulating the muscle protein synthesis pathway, independent of the systemic inflammatory response. In contrast to IGF-1, IL-15 not only directly promotes skeletal muscle protein synthesis but also inhibits muscle protein breakdown [70]. Busquets et al. confirmed that recombinant IL-15 (rIL-15) can mitigate muscle atrophy by reducing the total protein breakdown rate in isolated muscles (extensor digitorum longus) from COPD rats [71]. However, some studies have yielded conflicting results. In particular, Liu et al. reported that the IL-15 expression was increased in the diaphragm, intercostal muscles, and gastrocnemius muscles of COPD rats; moreover, the mRNA and protein levels of ubiquitin-conjugating enzyme E2 (14k), MAFbx, and Ub were significantly higher in the treatment group than in the control group. These molecular changes led to increased protein breakdown and decreased muscle weight in the respiratory muscles and diaphragm of COPD rats [42]. This discrepancy in the results might be attributed to the fact that the limited increase in IL-15 levels in COPD muscles is insufficient to counteract inflammation-induced muscle breakdown. This notion is supported by a previous cell experiment where recombinant TNF-α (rTNF-α) stimulated human myotubes to secrete IL-15 and alleviated inflammation-induced myotube atrophy. Co-culturing myotubes with rIL-15 and rTNF-α resulted in increased myotube thickness compared with TNF-α alone, suggesting the protective effect of the former against muscle atrophy [72]. In conclusion, rIL-15 treatment may offer an effective approach to ameliorate skeletal muscle atrophy in COPD. Dysregulation of IGF-1 and IL-15 highlights their crucial roles in maintaining muscle protein homeostasis and presents potential therapeutic targets for mitigating muscle atrophy in patients with COPD.

Myostatin exerts additional effects on muscle cell death by upregulating ferroptosis in COPD skeletal muscles, leading to muscle atrophy. Zhang et al. [73] found that the levels of glutathione peroxidase 4 and glutathione decreased in COPD skeletal muscles, whereas the expression levels of lipid peroxide and 4-hydroxynonenal were increased. Inhibition of myostatin binding to the hypoxia-inducible factor 2alpha (HIF2α) receptor resulted in partial restoration of glutathione peroxidase 4 and glutathione expression as well as simultaneous inhibition of ferroptosis. HIF2α, a key gene associated with ferroptosis in COPD, has been implicated in the pathogenesis of COPD by regulating immune cell infiltration and promoting inflammatory responses [74]. However, the precise mechanisms through which myostatin mediates ferroptosis by the HIF2α pathway in skeletal muscles in COPD remain unclear. Furthermore, its direct involvement in SMD in COPD is yet to be fully elucidated in further research.

### Myokines affect the regenerative capacity of skeletal muscles

Myokines hinder skeletal muscle regeneration by inhibiting the activity of SCs [75]. SCs are a heterogeneous population of stem and progenitor cells, which are essential for skeletal muscle growth, maintenance, and regeneration. They can express myogenic regulatory factors, including myogenic regulatory factor 5 (Myf5), myoblast determination protein 1 (MyoD), and myogenin [76]. In severe COPD patients with or without muscle atrophy, the expression levels of key muscle regeneration markers, such as Pax7, Myf5, MyoD, and myogenin, in the vastus lateralis (VL) muscles were decreased compared with those in the control group. Myostatin was significantly increased only in the VL of COPD patients with muscle atrophy [21]. Furthermore, in the VL of COPD patients with muscle atrophy, the number of activated SCs (Pax-7+/Myf-5-), CSA of the quadriceps femoris, and muscle strength were decreased [21]. Treatment with medium or high doses of the salidroside extract can potentially normalize myostatin expression in the gastrocnemius muscle of COPD rats. The treatment significantly alleviated the reduced expression levels of myogenin, Myf5, and myogenic regulatory factor 4 in the muscles of COPD rats, leading to improvements in skeletal muscle atrophy and muscle mass [69]. Polkey et al. observed that bimagrumab, a blocker of the activin type II receptor (a membrane receptor for myostatin), significantly increased the volume of thigh muscles in patients with COPD [77]. However, muscle function did not improve in COPD patients with low muscle mass, possibly due to the effect of myostatin loss on muscle regeneration function, leading to impaired muscle mass and function. To confirm the role of myostatin in promoting muscle regeneration, Yang et al. [78] conducted experiments where myostatin expression in SCs was either knocked out or blocked. Muscle regeneration was impaired in the absence of normal myostatin levels, emphasizing the importance of myostatin in activating SCs and promoting muscle regeneration.

Furthermore, chronic high IL-6 levels inhibit muscle differentiation and regeneration in COPD patients. Under physiological conditions, elevated IL-6 levels can activate the suppressor of cytokine signaling 3 (SOCS3) through the JAK2/STAT3 pathway and promote muscle myogenic differentiation. This activation stimulates the proliferation of myosatellite cells, thereby facilitating muscle regeneration. SOCS3 is induced via the JAK2/STAT3 pathway and acts as a negative regulator of STAT3-mediated signal transduction [79]. Gao et al. demonstrated that SOCS3 overexpression impaired myotube differentiation [80]. However, during chronic inflammation and muscle atrophy, STAT3-SOCS3 signaling is also activated in target skeletal muscles. In this context, the negative feedback loop mediated by IL-6 is disrupted, thereby inhibiting myoblast differentiation and decreasing the expression of myosin heavy chains [81]. This finding is consistent with the study by Bonetto et al. [82], where mice injected with recombinant IL-6 exhibited significant emaciation and notable weight loss. With prolonged exposure to IL-6, there was a progressive decrease in muscle weight. Another study revealed that low to moderate concentrations of IL-6 reduced the expression of MyoD and myogenin in C2C12 and primary human myoblasts, while normal to high concentrations of IL-6 significantly increased their expression [83]. These results suggest that IL-6-induced muscle differentiation is dependent on both time and concentration. Consequently, chronic IL-6 overexpression in patients with COPD may lead to the loss of the STAT3-SOCS3 negative feedback loop, thus inhibiting myoblast differentiation and impairing muscle regeneration. However, IL-6 knockout during differentiation of C2C12 myoblasts impairs the expression of the myogenic markers myogenin and myosin heavy chain IIb, thereby impairing myotube regeneration. Exogenous IL-6 administration partially restores fusion in IL-6 knockout cells [84]. These studies suggest that IL-6 is required for muscle regeneration and that lowering the IL-6 concentration to within physiological levels in COPD patients may increase muscle differentiation and thus promote muscle regeneration. Collectively, these findings provide new insights into the mechanisms that control the fate of skeletal muscle myoblast.

**Figure 1.**
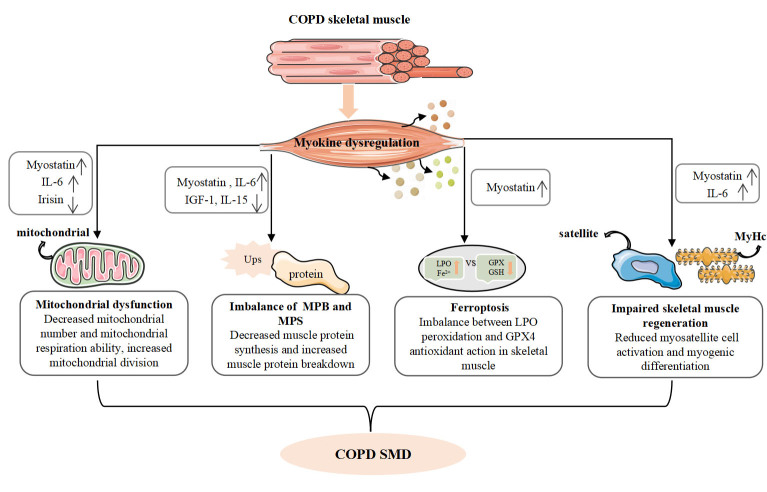
**Effect of myokines on SMD in COPD**. The expression of IL-6, IL-15, and myostatin was up-regulated, and the expression of irisin and IGF-1 was down-regulated in COPD skeletal muscle. (1) Up-regulated myostatin increases skeletal muscle mitochondrial division and promotes muscle atrophy by increasing skeletal muscle protein breakdown and ferroptosis, and blocks skeletal muscle regenerative capacity by inhibiting satellite cell activity. (2) Up-regulation of IL-6 decreases mitochondrial number, reduces mitochondrial respiratory capacity and skeletal muscle regenerative capacity, increases ubiquitination and breakdown of skeletal muscle; (3) Up-regulation of IL-15 inhibits skeletal muscle protein breakdown, but the increase of IL-15 expression in COPD is not enough to effectively inhibit the breakdown of COPD muscle; (4) Down-regulation of IGF-1 leads to the reduction of skeletal muscle protein synthesis by reducing the activation of PI3K-Akt axis; (5) Down-regulation of irisin may inhibit the protective effect on mitochondrial respiratory function by reducing P-MAPK levels. Abbreviations: COPD: chronic obstructive pulmonary disease; Drp1: dynamin-related protein 1; GPX4: glutathione peroxidase 4; GSH: glutathione; IL-6: interleukin-6; IL-15: interleukin-15; IGF-1: insulin-like growth factor-1; LPO: lipid peroxide; MyHc: myosin heavy chain; MPB: muscle protein breakdown; MPS: muscle protein synthesis; SMD: skeletal muscle dysfunction. ↓: down; ↑: up.

In conclusion, myokines play a significant role in the development and progression of SMD in COPD through their involvement in regulating mitochondrial function, disrupting the balance of skeletal muscle synthesis and breakdown, and interfering with the regenerative capacity of skeletal muscles (Fig. 1).

## ROLE OF EXERCISE IN INDUCING MYOKINE SECRETION IN SMD IN COPD

Exercise plays a pivotal role in COPD rehabilitation and serves as a valuable tool for preventing and ameliorating SMD. A randomized trial showed that muscle contraction inhibited the expression of myostatin mRNA and its downstream muscle atrophy factors MAFbx and MuRF1 mRNA. Furthermore, it protected the quadriceps mass compared with the muscle immobilization group [85]. Aerobic exercise increases irisin expression and ameliorates skeletal muscle atrophy and skeletal muscle cell apoptosis. Fndc5 knockout reduces aerobic exercise-induced anti-apoptotic and antioxidant capacity of cells, leading to skeletal muscle atrophy [86]. These results demonstrate that myokines mediate the benefits of exercise on SMD. The regulatory effects of exercise on various myokines in COPD as well as the potential underlying mechanisms are further discussed in the following text to identify potential target genes for the treatment of SMD in COPD.

### Effects of exercise on myokines in skeletal muscles in COPD

The role of exercise in regulating myokines is closely related to the exercise type. Resistance exercises decrease the myostatin levels in the quadriceps of patients with COPD and increase the myogenin/MyoD ratio, indicating an improvement in muscle synthesis and the restoration of muscle synthesis and breakdown balance [19]. Resistance and endurance exercises can also significantly increase the IGF-1 levels in COPD muscles; the latter upregulated myogenin mRNAs, Akt phosphorylation, glycogen synthase kinase-3β (GSK-3β), and mammalian target of rapamycin (mTOR) while decreasing the expression of forkhead box O1 to promote muscle hypertrophy and enhance muscle quality [87,88]. However, Costes et al. found that the phosphorylation levels of IGF-1 downstream factors Akt, GSK-3β, and 70-kDa ribosomal protein S6 kinase in the hypoxemic COPD group were lower than those in the normoxemic group after 8 weeks of endurance exercise; furthermore, the muscle CSA was significantly increased in the normoxemic group of COPD patients. Further *in vitro* analysis using C2C12 myotubes showed that hypoxia prevented the response of the Akt/mTOR and Akt/GSK-3β pathways to IGF-1 treatment [89]. Hence, hypoxemia may limit the positive effects of exercise on the skeletal muscles of patients with COPD. The effect of exercise on IL-6 remains unclear. Fischer et al. found that muscle IL-6 mRNA levels were significantly decreased in patients with COPD after endurance training, possibly due to an increase in the content of resting skeletal muscle glycogen, thereby inhibiting the IL-6 gene expression [90]. However, Ioannis et al. found that 10 weeks of resistance and endurance training had no significant effect on IL-6 mRNA expression in the lateral thigh muscles or the circulation of patients with COPD, but it significantly increased the IGF-1 mRNA levels [91,92]. In contrast, another study showed that resistance training significantly increased the IL-6 level in the skeletal muscles of patients with COPD but decreased its circulatory levels [93]. Hence, the increase in IL-6 secretion caused by muscle contraction may help alleviate the body’s inflammatory response, but further elucidation is needed in future studies. In summary, endurance training combined with resistance training may be superior to a single training modality due to the synergistic effect of both types.

The exercise duration influences the regulatory effect of exercise on myokines in COPD. In the study by Ljiri et al., the irisin levels in the skeletal muscles and serum of patients with severe COPD were significantly increased after 8 weeks of exercise; however, acute exercise did not affect the irisin level [13]. Another study found that despite the increased skeletal muscle CSA and 6MWD, 3-6 months of high-intensity cycling training did not significantly affect serum irisin levels in patients with COPD at different stages (the majority were in Global Initiative for Chronic Obstructive Lung Disease mild-to-moderate [I-II] stages; total of 20 patients) [17]. These discrepancies may be attributed to the varying severity of COPD, with exercise demonstrating a more pronounced effect on irisin in patients with severe COPD. Interestingly, Hubo et al. found that exercise increased the expression levels of Nrf2 and HO-1 by upregulating irisin in muscles, leading to an improvement in CS-induced COPD emphysema [94]. This finding provides new insights into the mechanism by which exercise improves SMD in COPD. Future experiments are needed to confirm whether irisin directly acts on Nrf2 in skeletal muscles to exert antioxidant effects. In their clinical trials, Constantin et al. [45] found that myostatin mRNA expression was significantly reduced at 24 h after acute exercise but was restored to the baseline value at 4 and 8 weeks in the COPD and healthy groups. In the absence of concurrent protein alterations, the transient decrease in myostatin mRNA expression induced by acute exercise may have limited physiological significance. The IGF-1 mRNA levels were increased after 10 weeks of exercise in both non-cachectic and cachectic patients with COPD. However, only non-cachectic patients exhibited an increase in IGF-1 protein levels, accompanied by a decrease in both myostatin mRNA and protein levels. This resulted in decreased expression of the myostatin downstream factors Atrogin-1 and MURF-1 in non-cachectic patients, leading to an increase in muscle CSA [95]. Limited research has investigated the regulatory effects of exercise on IL-15 in COPD skeletal muscles. A single study demonstrated that acute exercise increased the serum IL-15 levels in patients with COPD; meanwhile, long-term exercise for 12 weeks down-regulated serum IL-15 levels [93]. Therefore, the regulatory effects of exercise on IL-15 in patients with COPD may be duration-dependent. Further investigation is warranted to explore the specific regulatory effects of exercise. The abovementioned studies suggest that engaging in long-term exercise (≥8 weeks) can lead to a shift in muscle metabolism toward synthesis in COPD patients.

The effect of exercise intensity on different types of myokines is variable. High-intensity interval training can increase the IGF-1 expression levels and decrease the myostatin mRNA levels, thereby promoting muscle protein synthesis and inducing skeletal muscle hypertrophy [88]. In contrast, Micaela et al. found that low-intensity training, but not high-intensity interval training, led to beneficial effects in lowering myostatin concentrations. However, low-intensity training once a week had no effect on serum irisin levels in COPD patients [96]. Another clinical study found that moderate-intensity training significantly increased IGF-I mRNA and protein expression levels and significantly decreased myostatin levels in the plasma and muscles of patients with COPD, thereby increasing the 6MWD and muscle strength [91]. In summary, although low-intensity training can decrease myostatin levels, moderate-to-high-intensity training is more beneficial because the transient decrease in myostatin may have limited effects in improving SMD. Moderate- and high-intensity training can upregulate the expression of the muscle synthetic factors IGF-1 and irisin, promoting muscle hypertrophy and ameliorating skeletal muscle atrophy in COPD patients.

In conclusion, exercise can modulate levels of myokines and the expression of their downstream factors, thereby improving SMD in COPD. In particular, long-term (≥8 weeks) combined resistance and endurance training of moderate-to-high intensity has a pronounced effect on SMD. Such training can significantly increase IGF-1 and irisin levels, promoting muscle hypertrophy and improving muscle strength and function in COPD patients. However, the disease severity in COPD may attenuate or inhibit the modulatory effects of exercise on myokines, such as hypoxemia. Therefore, the benefits of exercise may vary according to individual patient variability. Furthermore, as myokines serve as mediators of exercise-induced improvement of SMD in COPD, many experiments are needed to delve into intricate variations in myokine modulation by exercise and to develop a detailed exercise protocol for COPD patients, including therapeutic approaches for myokines-based exercise. Studies on the effects of exercise on myokine modulation are shown in Table 2.

**Table 2:** Effects of exercise on myokines in patients with COPD.

Study	Species	Group(N)	Exercise style	Exercise program	Change of myokines	Skeletal muscle function indicators	Mechanisms of action on COPD
**Wajdi et al., 2014** [89]	COPD patients	COPD group (19)	Combined training	8 weeks, 3 day/w, 30 min of warm-up+45 min of resistance training	IGF-1, IGF-BP3↑ in serum	6MWD↑,FEV1%↑, FVC↑	/
**Costes et al., 2015** [90]	Normoxemic COPD patients, Hypoxemic COPD patients	COPD NG (15) COPD HG (8)	Combined training	8 weeks, 3 day/w, 20-30 min of endurance bicycle exercise+10-15 min of treadmill exercise	NG: no effect; HG: no effect	NCG: QMS↑, Wpeak↑, CSA↑; HCG: QMS↑, Wpeak↑	NG: p-Akt↑, p-GSK-3β↑, p-p70S6K↑; HG:/
**Vogiatzis et al., 2010** [96]	Non-cachectic COPD patients, Cachectic COPD patients	COPD CG (10) COPD NCG (19)	Combined training	10 weeks, IE: 30s work periods interspersed with 30s rest periods for 45 min/d CLE: mean intensity of 75 ± 5% Wpeak for 30 min/d	IGF-I mRNA↑ in CG and NCG; IGF-1 protein↑ in NCG; Myostatin mRNA and protein ↓ in NCG	CG: only CSA of type II fibers↑; NCG: CSA↑; Both: Wpeak↑,6MWD↑, MC/FR ↑	CG: activation of the NF-κB↓, Atrogin-1↑, MURF-1↑; NCG: activation of the NF-κB↓, Atrogin-1↓, MURF-1↓,MyoD mRNA↑
**Kubo et al., 2019** [95]	4 weeks old male mice	CS group (8) CSE group (8)	Cycle ergometers	12 weeks, 5 day/w, 3×10 min/day	CS: no effect; CSE: irisin mRNA↑,	CS: no effect; CE: alveolar apoptosis↓	Nrf2 and HO-1↑ (upregulation in the expression of antioxidant genes)
**Timm et al., 2014** [121]	Exacerbated hospitalized COPD patients	Control group (20) COPD WBVG (20)	Combined training	Control: 5 min mobilisation+ 5 min passive movement+ 10 min respiratory exercises WBV: 5 min mobilisation+ 5 min passive movement+ 10 min respiratory exercises complemented with sessions on the WBV device	Control: no effect; WBVG: irisin↑ in serum	Control: no effect; WBVG: 6MWD↑, CRT↓,	/
**Ijiri et al., 2015** [13]	Stable COPD patients	COPD AEG (8) COPD CEG (7)	Upright cycle ergometer	8 weeks, AEG: 3×10 min/day,1 day CEG: 3×10 min/day	AEG: no effect; CEG: Irisin↑ in serum	PAL↑ in both groups	/
**Despina et al., 2013** [45]	Stable stage with severe COPD	COPD group (59)	Resistance training	8 weeks, 3 sessions/w, 5×30 maximal knee extensions at an angular velocity of 180°/s	Myostatin mRNA↓ in muscle at 24h; No effect at 4 weeks and 8 weeks	Lean mass and strength in the thigh↑	Anabolic and transcription factor protein expression↑
**Thierry et al., 2010** [19]	Acute exacerbations of COPD	COPD RTG (17) Control group (19)	Resistance training	8 days, 3×8 resistance training of the quadriceps on a knee extension chair with an intensity of superior to 70% of the 1RM	Myostatin mRNA↓ in muscle	6MWD↑, QF↑	Myogenic signaling↑
**Ioannis et al., 2007** [92]	Stable COPD patients	COPD IEG (7) COPD CLEG (8)	Interval and constant-load training	10 weeks, 3 day\w, IEG: 100-130% Wpeak for 45 min CLEG: 65-75% Wpeak for 35 min	IGF-I mRNA↑ in muscle; IL-6: no effect	Wpeak↑, CSA↑	MyoD mRNA and protein↑ (skeletal muscle hypertrophy and regeneration↑).
**AlencarSilva et al., 2018** [94]	Stable patients with moderate to severe COPD	COPD EG (24) COPD MG (18)	Elastic resistance training; Conventional weight machines training	12 weeks, 3 day/w, 60 min/day, Flexion and extension training of upper limb muscles and knee joints lasting 15-30s each	IL-6 mRNA↑ in muscle skeletal muscle; IL-15 and IL-6 mRNA↓ in serum	Muscle strength↑	NF-κB↓, Systemic inflammation↓

AEG: acute exercise group; COPD: chronic obstructive pulmonary disease; CSA: cross-sectional area; CLE: constant-load exercise; CLEG: constant-load exercise group; CG: cachectic group; CS: cigarette smoking; CSE: CS+exercise; CRT: Chair-Rising-Test; CEG: chronic exercise group; EG: elastic band training group and elastic tube training group; FEV1%: the percentage of forced expiratory volume in one second shared by forced vital capacity; FVC: forced vital capacity; HG: hypoxemic group; HCs: healthy controls; IGF-1: insulin-like growth factor-1; ICF-BP3: Insulin-like Growth Factor-Binding Protein 3; IE: interval exercise; IEG: interval exercise group; IL-6: interleukin-6; IL-15: interleukin-15; 6MWD: 6-minute walk distance; MG/FR: muscle capillary/fiber ratio; MG: training group with weight machines equipment; MyoD: Recombinant Myogenic Differentiation; NG: normoxemic group; NCG: non-cachectic group; Nrf2: Nuclear factor erythroid 2-relatedfactor 2; NF-κB: Nuclear factor kappa-B; p-Akt: Phospho-AKT ; p-GSK-3β: phosho-GSK-3β; p-P706SK: phosho-p70S6K; PAL: physical activity level; QMS: quadriceps muscle strength; QF: quadriceps force; RTG: resistance training group; Wpeak: peak work-rate; WBVG: whole body vibration group; ↓: down; ↑: up.

### Key role of microRNAs for myokines in exercise

MicroRNAs (miRNAs) are small endogenous RNAs that regulate gene expression at the post-transcriptional stage. Their roles are established in both health and disease. Increasing clinical and experimental evidence suggests their involvement in the progression of chronic respiratory diseases, including COPD [97-99]. Aberrant expression of miRNAs in the respiratory muscles of COPD patients leads to decreased muscle endurance and strength, consequently affecting respiratory function and exacerbating disease progression [100]. Studies by Liu et al. have demonstrated that miRNAs can regulate muscle protein synthesis and degradation, mitochondrial function, and muscle regeneration, thereby influencing skeletal muscle function in COPD [101, 102]. Additionally, miRNAs are recognized as crucial molecules in regulating myokines by degrading mRNA or inhibiting gene translation, thereby exerting post-transcriptional control over muscle cell function [103-106]. Therefore, exercise may regulate the expression of myokine genes through miRNAs, thereby improving mitochondrial quality, increasing protein synthesis, and enhancing myocyte proliferation [107]. Lewis et al. [108] found that miR-1 expression in the skeletal muscles of patients with COPD was lower than that in the control group, and they found a negative correlation between miR-1 expression and IGF-1 mRNA and signaling. Exercise reduced the miR-1 expression level in skeletal muscles, upregulated the IGF-1 mRNA, and enhanced the metabolic signaling related to skeletal muscle synthesis [109]. Naz et al. found that the mi-29b levels were significantly elevated in COPD patients [110]. miR-29b targets IGF-1 and inhibits its expression [111]. In the *in vitro* and *in vivo* models of skeletal muscle atrophy, miR-29b overexpression reduced the IGF-1 protein levels, whereas knockout of the miR-29b gene or inhibiting its expression increased the IGF-1 expression in skeletal muscles [112]. Notably, exercise significantly down-regulated the miR-29b expression and activated the AKT/mTOR pathway to ameliorate angiotensin II-induced muscle atrophy [113]. miR-27a is significantly increased in the VL of COPD patients and is associated with muscle atrophy and muscle weakness [114]. Allen et al demonstrated that miR-27a targets myostatin [115]. Huang et al. also demonstrated that miR-27a over-expression promoted muscle cell proliferation by reducing myostatin levels in C2C12 cells [116].

As a crucial regulator of mitochondrial energy metabolism, PGC1-α plays a pivotal role in maintaining mitochondrial quality and acts as an upstream regulator of irisin [35,117]. However, few experimental studies have been conducted on upstream factors that regulate the PGC-1α/irisin axis. Queiroz et al. [118] applied gene sequence analysis to identify miR-696 as a novel suppressor of PGC-1α expression. miR-696 over-expression decreased PGC-1α expression and impaired mitochondrial function. These findings provide evidence for a mechanism by which miR-696 overexpression in skeletal muscle cells decreases PGC-1α expression and mitochondrial function. Aoi et al. [119] found elevated miR-696 levels and reduced PGC-1α levels in inactive mouse muscles. Interestingly, exercise interventions proved to be effective in reducing miR-696 expression and increasing the PGC-1α level. *In vitro* experiments further supported these findings, showing that inhibiting the miR-696 expression significantly upregulated the PGC-1α mtDNA and protein content and increased fatty acid oxidation. A clinical study on patients with COPD found that whole-body vibration training activated the PGC1-α expression in skeletal muscles, increased the irisin level, and induced mitochondrial biogenesis, thereby regulating muscle energy and protein metabolism [120,121]. Irisin also provides positive feedback to increase the PGC-1α expression, enhance uncoupling protein expression and mitochondrial biogenesis, and improve muscle function [122,123]. Exercise affects the PGC-1α/irisin pathway by inhibiting miR-696 expression, thereby upregulating the irisin expression in skeletal muscles in COPD.

**Figure 2.**
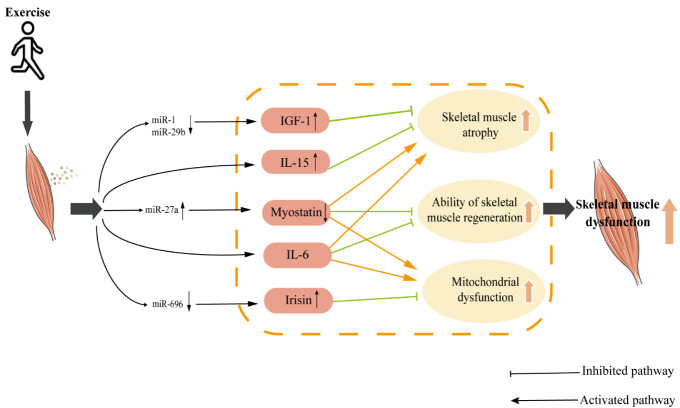
**Exercise improves the mechanism of myokines in SMD of COPD**. Exercise might regulate the expression level of myokines in skeletal muscle by: (1) Inhibiting expression of miR-1 and miR-29b up-regulates IGF-1 expression level; (2) Promoting expression of miR-27a down-regulates myostatin expression level; (3) may inhibiting expression of miR-696 up-regulates the irisin expression level. Abbreviations: IL-6: interleukin-6; IL-15: interleukin-15; IGF-1: insulin-like growth factor-1. ↓: down; ↑: up.

In conclusion, exercise holds potential to prevent the development of SMD in COPD by modulating the expression levels of key myokines through specific miRNAs. Exercise upregulates IGF-1 expression through miR-1 and miR-29b and influences myostatin and irisin expression through miR-27a and miR-696, respectively (Fig. 2). These miRNA-mediated pathways offer a promising avenue for improving SMD in COPD through exercise-induced regulation of myokine secretion. Further studies are needed to gain insight into the potential relationship between skeletal muscles, myokines, and miRNAs, which will provide new insights and directions for a more robust response to SMD in COPD.

## CHALLENGES AND FUTURE PERSPECTIVES

It is well recognized that the lack of exercise exacerbates SMD in COPD, whereas exercise has a positive effect on its improvement. Therefore, a growing amount of research is focused on unraveling the mechanisms by which exercise ameliorates SMD in COPD, particularly the molecular mechanisms underlying the prevention and treatment of SMD through exercise. In this context, myokines, which originate from muscles, seem to mediate the beneficial effects of exercise in improving SMD, thereby enhancing patients’ quality of life. Therefore, it is crucial to further elucidate the interactions among myokines and related signaling pathways using various exercise regimens. While current evidence indicates the significant role of myokines in regulating skeletal muscle function and acting as mediators in the improvement of SMD in COPD through exercise, the clinical application of myokines in treating SMD still faces significant challenges. Some issues include the controversy surrounding the correlation between serum concentrations of myokines and skeletal muscle parameters in COPD, lack of consistency between acute and chronic exercise responses, and translational challenges arising from differences between patients and exercise animal models. Moreover, the proposed potential therapeutic role of myokines in SMD in COPD is primarily based on information provided by animal experiments or a limited number of clinical trials. Therefore, more large-scale clinical trials are needed to evaluate and validate the aforementioned results and promote clinical translation.

To ascertain the therapeutic role of myokines in treating SMD in COPD and to determine their role in SMD treatment through exercise, several critical issues warrant further consideration. First, omics analyses (transcriptomics, proteomics, and metabolomics) should be performed to explore more myokines and determine appropriate concentrations for treating SMD in COPD. Additionally, employing bioinformatics-assisted omics techniques to investigate the crosstalk among myokines, and to determine the synergistic or inhibitory effects of various myokines, is crucial for improving the treatment of SMD in COPD. As the actions and biological effects of myokines become increasingly elucidated, they may be potentially harnessed to simulate the benefits of exercise for COPD individuals with limited exercise capacity or to counteract metabolic non-responsiveness or adverse reactions to exercise. Second, understanding the role of miRNAs in regulating the expression of myokines in the skeletal muscles of COPD patients is essential. As mentioned earlier, miRNAs regulate the gene expression of myokines, linking epigenetic events with myokines. However, a single miRNA can affect the translation of multiple mRNAs, and multiple miRNAs can affect the translation of a single mRNA. Therefore, gaining deeper insights into the potential relationships among COPD skeletal muscles, myokines, and miRNAs will provide new understanding and directions for addressing SMD in COPD. Third, quantifying and dynamically tracking myokines at different time intervals in the skeletal muscles of COPD patients in response to exercise, and determining the required exercise dose and type to induce positive outcomes in COPD, will effectively translate research into clinical treatment.

## CONCLUSIONS

SMD is a prevalent and significant issue in COPD and is characterized by skeletal muscle mitochondrial damage, muscle atrophy, and impaired activation of myosatellite cells. These factors collectively contribute to reduced muscle strength and endurance, which substantially affect the patient’s quality of life and prognosis. The pathological environment in patients with COPD leads to abnormal expression levels of myokines, which in turn disrupt skeletal muscle mitochondrial function, inhibit regenerative capacity, and promote muscle atrophy, leading to SMD. Exercise and modulation of myokines through exogenous supplementation or knockdown have significant potential in the prevention and treatment of SMD in COPD and offer valuable clinical implications. Exercise modulates the expression levels of myokines, a process dependent on factors such as exercise type, exercise duration, and exercise intensity. Notably, long-term (≥8 weeks) combined resistance and endurance training of moderate-to-high intensity has a pronounced effect on increasing the levels of IGF-1 and irisin in COPD skeletal muscle, promoting muscle hypertrophy and improving muscle strength and function. These beneficial effects of exercise are mediated, at least in part, by miRNAs, which play a regulatory role in the expression of myokine genes during exercise. miR-1 and miR-29b are implicated in the regulation of IGF-1 gene expression during exercise, while miR-27a and miR-696 are involved in the regulation of myostatin and irisin gene expression, respectively. To the best of our knowledge, this study represents the first comprehensive discussion on the role of myokines in the development of SMD in COPD. The results reveal novel therapeutic targets within the field of COPD rehabilitation. Furthermore, targeted regulation of irisin, IGF-1, IL-15, and myostatin-related genes may offer promising strategies for the prevention and treatment of COPD in the future. In conclusion, the study of myokines has provided new insights into the pathogenesis of SMD in COPD. While there is currently insufficient data to predict prognosis and treatment response to current therapies, myokines hold promise as candidate biomarkers for investigating disease phenotypes and identifying potential novel therapeutic targets.
